# Long-term metagenomic insights into the roles of antiviral defense systems in stabilizing activated sludge bacterial communities

**DOI:** 10.1093/ismejo/wraf051

**Published:** 2025-03-17

**Authors:** Qifeng Zhang, Jie Li, Jinhua Tuo, Shengnan Liu, Yang Liu, Peng Liu, Lin Ye, Xu-Xiang Zhang

**Affiliations:** State Key Laboratory of Pollution Control and Resource Reuse, School of Environment, Nanjing University, Nanjing 210023, China; State Key Laboratory of Pollution Control and Resource Reuse, School of Environment, Nanjing University, Nanjing 210023, China; State Key Laboratory of Pollution Control and Resource Reuse, School of Environment, Nanjing University, Nanjing 210023, China; State Key Laboratory of Pollution Control and Resource Reuse, School of Environment, Nanjing University, Nanjing 210023, China; State Key Laboratory of Pollution Control and Resource Reuse, School of Environment, Nanjing University, Nanjing 210023, China; State Key Laboratory of Pollution Control and Resource Reuse, School of Environment, Nanjing University, Nanjing 210023, China; State Key Laboratory of Pollution Control and Resource Reuse, School of Environment, Nanjing University, Nanjing 210023, China; State Key Laboratory of Pollution Control and Resource Reuse, School of Environment, Nanjing University, Nanjing 210023, China

**Keywords:** bacteriophages, defense systems, microbial communities, pan-immunity, activated sludge

## Abstract

Bacteria have evolved various antiviral defense systems (DSs) to protect themselves, but how DSs respond to the variation of bacteriophages in complex bacterial communities and whether DSs function effectively in maintaining the stability of bacterial community structure and function remain unknown. Here, we conducted a long-term metagenomic investigation on the composition of bacterial and phage communities of monthly collected activated sludge (AS) samples from two full-scale wastewater treatment plants over 6 years and found that DSs were widespread in AS, with 91.1% of metagenome-assembled genomes (MAGs) having more than one complete DS. The stability of the bacterial community was maintained under the fluctuations of the phage community, and DS abundance and phage abundance were strongly positively correlated; there was a 0–3-month time lag in the responses of DSs to phage fluctuations. The rapid turnover of clustered regularly interspaced short palindromic repeat spacer repertoires further highlighted the dynamic nature of bacterial defense mechanisms. A pan-immunity phenomenon was also observed, with nearly identical MAGs showing significant differences in DS composition, which contributed to community stability at the species level. This study provides novel insights into the complexity of phage–bacteria interactions in complex bacterial communities and reveals the key roles of DSs in stabilizing bacterial community structure and function.

## Introduction

Bacteriophages are ubiquitous in both natural and artificial environments, and they are the most numerous biological entities on Earth [1–4], with an estimated total number approaching 10^31^ [5]. In contrast to bottom-up drivers (e.g. temperature, oxygen levels, and nutrient availability), phages are considered major top-down drivers (predatory) of microbial communities [6, 7], as they can cause the lysis of 20%–40% of bacterial populations in various environments [8, 9]. This has motivated interest in the use of phages to manipulate microbial communities and functions in clinical and engineering applications [10–13].

In contrast to simple communities consisting of a few species, which are highly sensitive to the effects of phages, the addition of phages to complex communities sometimes only reduces the overall bacterial abundance without significantly affecting community structure and function [14–18]. Moreover, the coexistence of highly dynamic phage communities with relatively stable bacterial communities has been observed in marine [19] and anaerobic digesters [20]. These findings suggest that complex communities may be more resistant to the reintroducing of lytic phages, sparking critical discussions about the role of phages in shaping bacterial communities within complex ecosystems [21]. However, the underlying mechanisms remain unclear. This is an important issue to address, whether the goal is to overcome this resistance (e.g. in phage therapy) or to exploit it (e.g. in designing synthetic microbial community with enhanced phage resistance).

Prokaryotic antiviral defense systems (DSs) provide a potential explanation for bacterial community resistance to phages [22]. Since 2018, using the “guilt-by-association” approach within defense islands, many new DSs have been identified, in addition to the well-known adaptive DS clustered regularly interspaced short palindromic repeats (CRISPR)-Cas, innate DS restriction-modification (RM), and abortive infection systems [23]. This has expanded the catalog of known DSs to include hundreds of types [24]. The growing DS databases permit uncovering the composition of DSs in various complex environments including soil, marine, human gut [25], drinking water [26], and cheese [27]. Despite the widespread of DSs, their functions in maintaining the stability of complex bacterial community remain implicit due to the lack of direct observations in phage–bacteria dynamic interactions. We speculate that DS abundance should track with phage abundance fluctuations and the adaptive DS CRISPR-Cas immune repertoires being updated in response to phage dynamics if DSs works in stabilizing complex bacterial communities. Therefore, it is necessary to conduct ecological-scale time-series studies of DS, particularly focusing on the dynamics of DS along with their associated phage and bacterial communities, to deepen our understanding of the ecological significance of DS.

The phenomenon of “pan-immunity” in the composition of DSs has been observed in *Escherichia coli* [28], *Pseudomonas aeruginosa* [29], and cheese-associated bacteria [27], where different strains of the same species exhibit distinct DS profiles. This unique phenomenon is thought to result from the long-standing trade-off between phage resistance and adaptive costs [30]. A plausible hypothesis posits that the ecological benefit of pan-immunity within a community lies in ensuring the persistence of bacteria at the species level [30]. Specifically, the dominance of similar strains with different levels of phage resistance (and corresponding adaptive costs) varied depending on the balance between phage pressure and adaptive costs in the environment, which ensured that some members of the species survived under variable phage pressures. According to this hypothesis, the relatively stable species composition in bacterial communities may conceal fluctuations at the strain level, which would also require time-series studies to validate.

In this study, we selected the activated sludge (AS) system from wastewater treatment plants (WWTPs) as our research subject. AS consists of a semi-controlled engineered microbial community [31], where WWTPs regulate bottom-up drivers such as dissolved oxygen (DO), pH, and hydraulic retention time (HRT) [32]. This makes AS more stable compared to other complex microbial systems, positioning it as an ideal model for studying bacteria–phage interactions [2]. Specifically, we conducted 6 years of sampling on AS from two full-scale WWTPs located in Nanjing, Jiangsu Province, China. Using a metagenomic approach, we characterized the long-term dynamics of bacterial and phage communities. We then explored the diversity of DSs and their bacterial hosts in the AS by identifying complete DSs and analyzing their genetic characteristics. The relationship between the changes in phage abundance and DS abundance over long time scales was characterized using the catalog of AS DSs. A comparative genomic analysis was also conducted to clarify the potential role of pan-immunity among similar metagenome-assembled genomes (MAGs) in facilitating the rapid turnover of DSs. Overall, our findings highlight the critical role of DSs in stabilize complex communities and provide new insights into the complexity of phage–bacteria interactions.

## Materials and methods

### Sample collection, DNA extraction, and metagenomic sequencing

AS was sampled from two municipal WWTPs located in Nanjing, Jiangsu, China. Dachang WWTP (WWTP-DC) uses the oxidation ditch process with a treatment capacity of 100 000 m^3^/d and serves an area of 38.3 km^2^. Jiangxinzhou WWTP (WWTP-JXZ) uses the anaerobic-anoxic-oxic process with a treatment capacity of 670 000 m^3^/d and serves an area of 98.0 km^2^. From January 2013 to September 2018, 50 ml of AS samples were collected monthly from aerobic tanks. Given that the viruses recovered by the viral-like particle-concentrated metagenomic approach in AS are mainly eukaryotic viruses (particularly those infecting vertebrates), phages are the main taxa recovered via non-concentrated metagenomic approaches [33]. We thus used the non-concentrated metagenomic approach to investigate phage community dynamics in AS. Immediately after collection, the samples were fixed with 100% ethanol at a ratio of 1:1 (v/v) for biomass fixation [34]. Total DNA was extracted using a FastDNA Spin Kit for Soil (MP Biomedicals, CA, USA).

Metagenomic sequencing was performed on the HiSeq X Ten System (PE150 strategy, 350 bp insert size) (Illumina, USA), and 134 datasets (69 from WWTP-DC and 65 from WWTP-JXZ; 4 samples from WWTP-JXZ failed the quality control test) and 1564 Gb were obtained. The details of each dataset are shown in Supplementary Table S1.

### Public dataset collection

In addition to the 134 AS metagenomes sequenced from the two WWTPs in this study, we downloaded a 9-year time-series metagenomic dataset from the Shatin WWTP in Hong Kong and processed it using the same bioinformatics workflow as used on our data. Similar to the Nanjing dataset, the Hong Kong AS dataset was also generated by paired-end sequencing on the HiSeq System (Illumina, USA). Sample collection and DNA extraction were performed using the same protocol, including biomass fixation with 100% ethanol, enrichment by centrifugation, and DNA extraction using the FastDNA Spin Kit for Soil [34].

### Metagenomic assembly and metagenome-assembled genomes

The sequencing and downloaded metagenomic raw data were subjected to quality control and trimming using KneadData v0.12.0 (https://github.com/biobakery/kneaddata) with built-in FastQC v0.12.1 (https://github.com/s-andrews/FastQC) and Trimmomatic v0.39 [35] to obtain high-quality clean data. The clean data were then assembled using MEGAHIT v1.2.9 [36] with default parameters, and contigs with length ≥ 1000 bp were retained. CoverM v1.0.12 [37] with the built-in BWA-MEM v0.7.17 [38] indicated that 56.6% ± 9.9% of the reads in each sample could be mapped to these contigs with an identity ≥95%. Prodigal v2.6.3 [39] was then used to predict the open reading frames (ORFs) of the contigs.

Contigs ≥1000 bp were input into the binning module of metaWRAP v1.3.2 [40] (-metabat2 -maxbin2 -concoct) to recover MAGs. The completeness and contamination of MAGs were assessed using CheckM v1.0.12 [37], and only the MAGs with completeness ≥90% and contamination ≤10% were retained for downstream analysis. GTDB-Tk v2.3.2 [41] was used to annotate the species of MAGs based on marker genes. FastANI v1.34 [42] was used to calculate the average nucleotide identity (ANI) between all possible pairs of MAGs. Finally, a phylogenetic tree of the MAGs was constructed using PhyloPhlAn v3.1.1 [43].

### Dynamics of the microbial community

For the bacterial community, taxonomic annotation of the clean reads was performed using Kraken2 v2.1.3 [44] with default parameters. Abundance calculations were then conducted using Bracken v2.8 [45] to obtain species abundance tables for the bacteria. At the strain level, we used StrainPhlAn v4.1.1 [46] to reconstruct the consensus strain sequences in metagenomic samples and constructed a phylogenetic tree of the consensus strain using PhyloPhlAn v3.1.1 [43] to compare differences in the consensus strains in metagenomic samples.

For the phage community, contig datasets were initially screened for viral contigs (VCs) using VirSorter2 v2.2.4 [47] and VIBRANT v1.2.1 [48]. For contigs ≥5000 bp, VCs were identified based on the following criteria: (i) VirSorter2 score ≥ 0.9; (ii) VirSorter2 score ≥ 0.7 with hallmark genes; and (iii) VirSorter2 score ≥ 0.5 and validated by VIBRANT. Subsequently, the quality of these VCs was assessed, and host contamination was removed by CheckV v2.3.1 [49]. High-confidence VCs for downstream analysis were then selected using the following criteria: (i) completeness ≥50%; (ii) the number of viral genes − 5 * host genes > 0. All high-confidence VCs were clustered into species-level viral operational taxonomic units (vOTUs) using CD-Hit v2.3.1 [50] at 95% identity and 85% alignment coverage (relative to the shorter sequence) [51]. Finally, vOTU abundances were calculated using the CoverM v0.6.1 (https://github.com/wwood/CoverM) pipeline with BWA-MEM v0.7.17 [38] in contig mode, with parameters set to identity ≥95% and coverage ≥75%, resulting in reads per kilobase per million mapped reads (RPKM) values for the vOTUs.

To assess the functionality of the bacterial community, we used HUMAnN3 v3.9 [52] to map metagenomic reads to the UniRef90 database v201901b [53] and calculate the abundance of each gene family. We then quantified the functional abundance of metagenomic samples by associating UniRef90 with MetaCyc v24 [54] pathways.

### Identification and quantification of prokaryotic antiviral defense systems

DSs in contigs and MAGs were identified using DefenseFinder v1.2.1 [24]. Given that a single defense gene might not be effective for resisting phages, only complete DSs were considered in downstream analysis. The abundance of DSs was determined by the abundance of contigs containing complete DSs [26]. DS contig abundances were calculated using the CoverM v0.6.1 (https://github.com/wwood/CoverM) with BWA-MEM v0.7.17 [38] in contig mode (identity ≥95% and coverage ≥75%), which yielded RPKM values for the DS contigs.

### Detection of clustered regularly interspaced short palindromic repeat arrays in metagenomic reads and target mapping

CRISPR arrays were identified in the clean reads using CRASS v1.0.1 [55]. Next, spacers were extracted from the clean reads using the extract module of crisprtools v1.0.1 [55]. Redundancy in the spacers was removed using CD-Hit v2.3.1 [50] with parameters set to 100% identity and 100% alignment coverage (relative to the shorter sequence). Spacer density was recorded as the number of spacers per million reads.

To determine the targeting rate of metagenomic read spacers against vOTUs, BLASTn v2.3.1 [56] was used to align spacers to vOTUs with ≥95% identity and ≤1 mismatch.

### Identification of mobile genetic elements (MGEs)

MGEs in the contig datasets were identified using mobileOG-db beatrix-1.6 [57]. ORFs were aligned to MGE sequences using BLASTn v2.3.1 [56] with ≥75% identity and ≥75% coverage.

### Determination of environmental variables

While collecting monthly metagenomic samples, we also recorded the water physicochemical properties and process parameters of WWTP-DC, including temperature (T), DO, pH, chemical oxygen demand, biochemical oxygen demand (BOD), suspended solids, NH_4_-N, NO_3_-N, total nitrogen, total phosphorus, sludge settling velocity, sludge volume index, HRT, and sludge retention time (SRT) were determined with the standard water and wastewater monitoring and analysis methods of China [58], as shown in Supplementary Table S2.

### Statistical analyses

All statistical analyses were performed and graphs were made using R v4.3.0 and Origin 2023. The Bray–Curtis distance for community analysis was calculated using the vegan package. Phylogenetic trees were visualized using the ggtree and ggplot2 packages.

## Results

### Bacterial community remained stable under fluctuations in the phage community over 6 years

To investigate the long-term succession dynamics of microbial communities, we conducted monthly sampling for 6 years and metagenomic sequencing of the AS from WWTP-DC and WWTP-JXZ in Nanjing, China. We recovered 2575 VCs using various viral prediction tools, and they were further clustered at the species level (ANI ≥ 95%), which resulted in 1991 vOTUs to determine the species composition of the phage communities.

**Figure 1:**
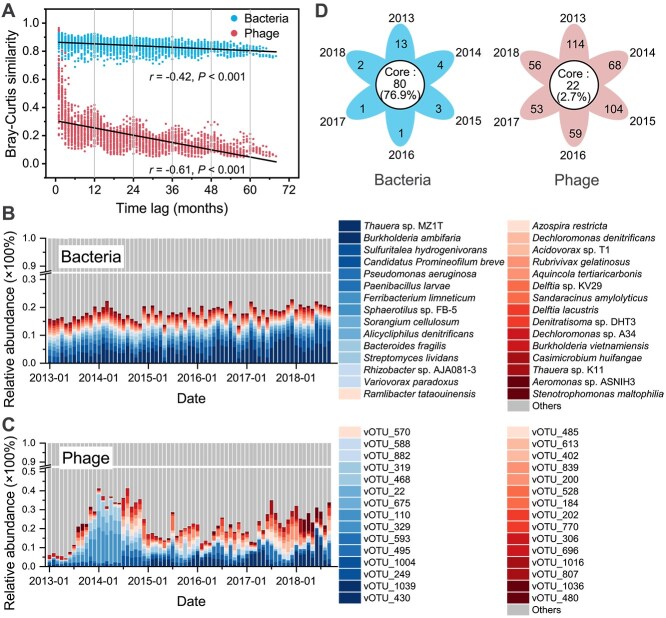
Temporal dynamics of the bacterial and phage community. (A) Bray–Curtis similarity of bacterial and phage communities over different time lags, with each point representing a sample pair, encompassing all possible sample pairs (*n* = 2346). (B) Relative abundance changes in the species of bacterial communities, showing the 30 most abundant species. (C) Relative abundance changes in the species of phage communities, showing the 30 most abundant species. (D) Annual distribution of unique and core species in bacterial and phage communities. Species with an average relative abundance of more than 0.1% in each of the 6 years were defined as core species, and species with an average relative abundance of more than 0.1% in only 1 year were defined as unique species. *Note*: This figure only presents data from WWTP-DC, and those of WWTP-JXZ are presented in the Supplementary Materials (Supplementary Fig. S1).

Over the 6 years, the bacterial communities exhibited high temporal stability under fluctuations in phage communities in both WWTPs. The Bray–Curtis similarity of the bacterial community consistently remained above 0.80, whereas that of the phage communities rapidly decreased to 0.23 within each 6-month interval, which reflects significant successional changes (Fig. 1A and Supplementary Fig. S1A). The Bray–Curtis similarity of the phage communities showed a sinusoid-like pattern with 12-month intervals, indicating pronounced seasonal variation. Moreover, the functionality of the bacterial community exhibits similar temporal stability as the community structure (Supplementary Fig. S2).

Specifically, species abundance within the bacterial communities showed minor fluctuations over time, with few instances of species extinction or the emergence of new species (Fig. 1B and C and Supplementary Fig. S1B and C). In other words, a persistent set of bacterial communities was maintained over the 6 years. Using WWTP-DC as an example, the core community comprised 80 species, which was significantly more than the number of unique species in each year (4 ± 4.2 species/year) (Fig. 1D). Among these core species, *Thauera* sp. MZ1T (*Thauera aminoaromatica*) was the most abundant species in both WWTPs (2.5% ± 1.3% in WWTP-DC and 2.4% ± 1.4% in WWTP-JXZ). In contrast, only 22 core phage species persisted over the 6 years, indicating that there was frequent turnover (i.e. lack of stability) in the phage communities (Fig. 1D). For example, in WWTP-DC, the relative abundance of vOTU_329 rapidly increased from 1.4% to 9.4% over 6 months (July 2013 to January 2014), making it the most abundant phage, and then declined sharply to 0.3% over the next 6 months (January 2014 to July 2014); its low abundance was maintained until it completely disappeared after February 2017.

### Bacteria harbored diverse antiviral defense systems in activated sludge

**Figure 2:**
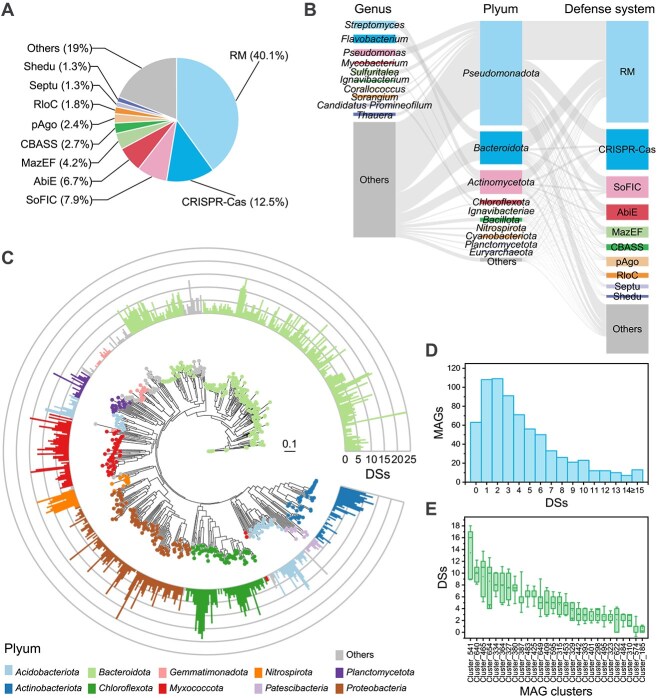
Composition profiles of DSs in AS. (A) Abundance distribution of DS types. (B) Composition of DS carriers at the phylum and genus levels. (C) Number of DSs in AS MAGs (*n* = 705). The inner ring shows the phylogenetic tree of MAGs, and the outer ring is a bar chart showing the number of DSs, with different colors indicating different phyla. Only the 10 most abundant phyla are labeled. (D) Distribution of the number of DSs in MAGs. (E) Statistics of the number of DSs in MAGs across different MAG_clusters (ANI ≥ 95%); only the MAG_clusters with more than five members are listed.

To reveal the potential role of bacterial antiviral DSs in maintaining the stability of bacterial communities, we analyzed the distribution of DSs and their bacterial hosts. At the contig level, a total of 39 747 complete DSs (including 72 350 ORFs) belonging to 111 types and 180 subtypes were detected in 0.3% of all contigs, among which RM (40.1%), CRISPR-Cas (12.5%), standalone protein with Fic domain (SoFIC) (7.9%), and abortive infection E (AbiE) (6.7%) were the most abundant DSs in the AS (Fig. 2A). Taxonomic annotation of DS-carrying contigs revealed that these DSs were distributed across 33 phyla and 939 genera (Fig. 2B), covering 98.8% of the phyla and 91.0% of the genera within the bacterial communities, revealing the phylogenetic university of DSs in AS. Furthermore, searching complete DSs in the entire MAG dataset of AS (*n* = 705) revealed that 91.1% of the MAGs harbored ≥1 DSs, with an average of 4.32 DSs per MAG (Fig. 2C), and the DS numbers in AS MAGs exhibited a binomial distribution (Fig. 2D). MAG_44 (f_*Saprospiraceae*) was found to carry the highest number (25) of DSs.

We also observed a clustering pattern of the number of DSs in the phylogenetic tree, wherein phylogenetically similar MAGs tended to have similar numbers of DSs. To explore the relationship between phylogeny and DSs, we clustered MAGs with ANI ≥ 95% into MAG_clusters (representing species-level clusters) and analyzed the number and composition of DSs in MAG_clusters with more than five members. The results revealed significant differences in the number of DSs among different MAG_clusters (Fig. 2E), ranging from 0.71 ± 0.45 DSs/MAG in Cluster_185 (f_*GWC2–71-9*) to 13.44 ± 4.30 DSs/MAG in Cluster_541 (g_*SSC4*). Moreover, bacterial species exhibited taxon-specific DS repertoires (Supplementary Fig. S3), indicating that different species tended to recruit different DSs. RM and CRISPR-Cas systems were the most common DSs within these taxon-specific DS repertoires, only 8.0% and 12.0% of MAG_clusters were lack in RM and CRISPR-Cas in all members, respectively, which is consistent with their high abundances (Fig. 2A).

### Antiviral defense systems responded rapidly to the fluctuations in the phage community

**Figure 3:**
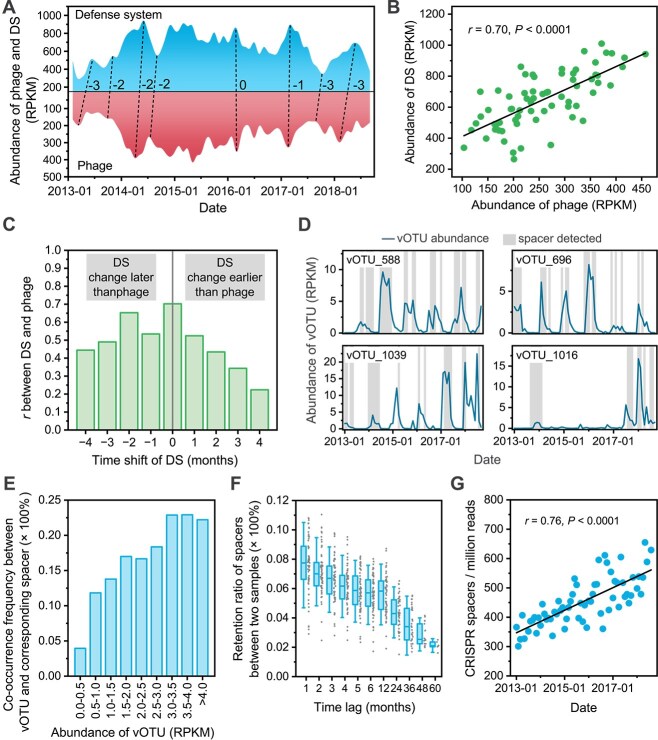
Temporal dynamics of DSs in AS. (A) Temporal changes in DS and phage abundances. (B) Linear regression between DS abundance and phage abundance. (C) Time-shift correlation between DS abundance and phage abundance. The *x*-axis values indicate the time shift of the DS; e.g. −1 represents the correlation between the phage abundance of each month and the DS abundance of the following month. (D) Co-occurrence between vOTUs and their corresponding spacers. The line represents the abundance of vOTUs, and the gray background indicates the presence of spacers targeting the vOTU at that time point. (E) Co-occurrence frequency between vOTUs in different RPKM ranges and their corresponding spacers. (F) Retention ratio of CRISPR spacers at different time lags, with each point representing a sample pair. (G) Linear regression between CRISPR spacer density and time. *Note*: This figure only presents the data from WWTP-DC, and those of WWTP-JXZ are presented in Supplementary Materials (Supplementary Figs S4, S7, and S9).

To clarify the response of bacterial DSs to fluctuations in the phage community, we compared the total abundance of DSs with phage abundances (Fig. 3A and Supplementary Fig. S4A). Over the 6 years, the abundance of DSs and phages in both WWTPs were significantly positively correlated (WWTP-DC: *r* = 0.70, *P* < .0001; WWTP-JXZ: *r* = 0.63, *P* < .0001) (Fig. 3B and Supplementary Fig. S4B). Meanwhile, we also reanalyzed a public metagenomic data of 9-year AS samples sampled from the Shatin WWTP in Hong Kong and found similar patterns during its first three stable years of operation (after the third year, periodic NaClO addition to control sludge foaming significantly altered community structure [6, 59]) (*r* = 0.74, *P* < .0001) (Supplementary Fig. S5). Specifically, among the 10 most abundant DSs (accounting for 81.0% of all DSs), nine were significantly positively correlated with phage abundance (*P* < .01), with the exception of MazEF (Supplementary Fig. S6). The peaks and troughs in DS abundance lagged behind those of phage abundance by 0–3 months (Fig. 3A and Supplementary Fig. S4A). This was further confirmed by the time-shift result that the correlations decayed more slowly when DS abundance was shifted backward in time, indicating that changes in DS abundance tended to follow those of phage abundance (Fig. 3C and Supplementary Fig. S7). Additionally, we analyzed the correlation between DS abundance and 14 water physicochemical properties and process parameters (Supplementary Table S2) and found a small but significantly positive correlation between BOD and DS abundance (*r* = 0.32, *P* < .01) (Supplementary Fig. S8).

In addition to the response in the total abundance of DSs, we also observed genomic evidence of the rapid bacterial response to phage community fluctuations, as revealed by adaptive DS CRISPR-Cas in AS. We extracted CRISPR spacers from metagenomic reads and used BLAST to match these spacer sequences with vOTU sequences. Spacers targeted 40.8% (WWTP-DC) and 35.6% (WWTP-JXZ) of the vOTUs, and a co-occurrence pattern was observed for the increase in abundance of any vOTU and the presence of corresponding spacers (Fig. 3D). Specifically, the co-occurrence probability increased from 3.9% for vOTUs with 0 ≤ RPKM ≤ 0.5% to 22.2% for vOTUs with RPKM > 4.0 (Fig. 3E). When the abundance of a vOTU decreased, the corresponding spacers were quickly discarded rather than being retained. A longer time lag of sample collection seemed to result in a greater difference in spacer repertoires. Even for samples from consecutive months, the retention rates of spacers were only 7.6% ± 1.9% (WWTP-DC) and 3.7% ± 1.1% (WWTP-JXZ) (Fig. 3F and Supplementary Fig. S9A), indicating the continuous rapid turnover of CRISPR spacer repertoires. The density of CRISPR spacers increased significantly over time in both WWTPs (WWTP-DC: *r* = 0.76, *P* < .0001; WWTP-JXZ: *r* = 0.68, *P* < .0001), with CRISPR spacer density increasing by 55.8% in WWTP-DC and 25.4% in WWTP-JXZ over the 6 years (Fig. 3G and Supplementary Fig. S9B). The rapid turnover of CRISPR spacer repertoires suggests that the annual increase in spacer density did not stem from their accumulation.

### Pan-immunity played a crucial role in stabilizing the bacterial communities at the species level

**Figure 4:**
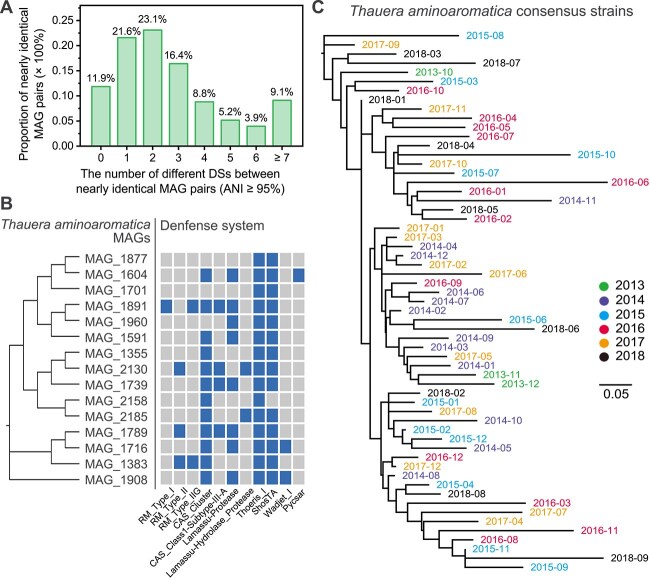
Pan-immunity of DSs in AS. (A) The number of different DSs in nearly identical MAGs, encompassing all possible ANI ≥ 95% MAG pairs (*n* = 329). The *y*-axis is the proportion of MAG pairs with the corresponding number of DS differences to the total number of MAG pairs. (B) Composition of DSs in MAGs annotated as *Thauera aminoaromatica* according to GTDB (*n* = 15, ANI ≥ 95%), with the phylogenetic tree of MAGs on the left. (C) Consensus strains (*n* = 59) of *T. aminoaromatica* constructed from metagenomic data using StrainPhlAn, with labels colored by year.

To test the hypothesis of pan-immunity in AS, we counted the number of DS differences between all ANI ≥ 95% MAG pairs (*n* = 329), excluding those MAGs without DSs to quantify the differences in DS composition among similar MAGs. Only 11.9% of MAG pairs had the same DS composition, and 43.4% of the MAG pairs differed in three or more DSs (Fig. 4A). For example, MAG_cluster_315 (s_*T. aminoaromatica*, the most abundant species in both WWTPs) comprised 15 MAGs with ANI ≥ 95% and contained 11 different DSs; however, each MAG carried only 4.27 ± 1.53 of these DSs (Fig. 4B). This indicates that even nearly identical MAGs have diverse DS compositions.

We further analyzed variations of bacterial strain-level diversity associated with stable species-level communities. Given the constraints of metagenomic sequencing, we could not track strain abundance with the same precision as species abundance. Instead, we identified differences in strain composition within a specific species by reconstructing consensus strain sequences. Here, we used StrainPhlAn [46] to successfully reconstruct the consensus strain sequences of *T. aminoaromatica* in 59 out of 69 samples from WWTP-DC and constructed a phylogenetic tree (Fig. 4C). As expected, except for a few adjacent samples with similar consensus strains (e.g. April and May 2016), the consensus strains of *T. aminoaromatica* significantly differed among most of the samples, indicating that there were strong fluctuations at the strain level.

## Discussion

Bacteria–phage interactions have long been a key topic in microbial ecology. Our longitudinal sampling of AS systems from the two WWTPs demonstrated that bacterial communities can remain relatively stable despite fluctuations in phage communities. This is consistent with increasing evidence that bacteria–phage interactions in complex communities do not follow the same patterns observed in model experimental communities [21, 60, 61].

The widespread distribution of DSs in AS system offers a potential explanation for the temporal stability of bacterial communities. Similar to findings in marine environments, soil, and the human gut [25], most bacterial MAGs in the AS harbor more than one complete DS. This suggests that phage resistance may be a fundamental function of bacterial communities. However, the composition of these DSs varies across environments. For example, SoFIC is the third most abundant DS in AS, marine, and soil environments, but it ranks 15th in the human gut [25]. This discrepancy may be attributed to the taxon-specific of most DSs. Specifically, we found that, except for the RM and CRISPR-Cas present in most MAG clusters in AS, the distribution of other DSs is influenced by phylogeny. An analysis based on complete bacterial genomes from NCBI RefSeq similarly shows that, apart from RM (80%) and CRISPR-Cas (39%), the frequencies of other DSs are all below 20%, and some DSs are enriched only in specific phyla (such as BstA and RexAB are only present in *Proteobacteria*) [24]. These results suggest that different environments, by recruiting distinct bacterial communities, likely lead to variations in DS composition. This also explains why RM and CRISPR-Cas are the two most abundant DSs across various environments, as they are non-taxon-specific DSs, making them less susceptible to the species composition differences of the environments.

We observed a rapid response of DS abundance to phage abundance in AS systems in different wastewater treatment processes. Previous studies have shown that the number of DSs at the individual bacterial level determines phage resistance [29], whereas the total abundance of DS at the community level may be a macroscopic reflection of the changes in the average DS number in individual genomes as phage stress changes. Moreover, variation in DS abundance was not dominated by a single DS type, and nearly all the high-abundance DSs were significantly positively correlated with phage abundance. This is likely because most DSs can only counter specific phages; consequently, the cooperative action of multiple DSs is necessary for combating diverse phage communities [22].

CRISPR-Cas, the only known adaptive DS in prokaryotes, can remember, recognize, and target foreign nucleic acids [62]. By matching CRISPR spacers with phage sequences, we identified a rapid memory response to emerging phages, which provides another dimension of dynamic data beyond the abundance dynamics of DSs and phages, offering more direct and non-correlative genomic evidence. Although this does not fully prove the functionality of the CRISPR spacer repertoires, it undoubtedly indicates that they are under selection [27, 63]. Besides, CRISPR-Cas appears to be an exception to the pattern of DS abundance changes, which was more strongly correlated with time than with phage abundance (Supplementary Fig. S10). Additionally, the density of CRISPR spacers increased significantly over time. This can be attributed to the relatively broad spectrum of CRISPR-Cas targets. Although CRISPR does not provide complete immunity against all phages, it has a broader defense range compared with other systems. Previous studies have revealed that CRISPR spacers in cheese-related communities target nearly 50% of cheese phages [27]. The results of our study showed that they targeted 40.8% (WWTP-DC) and 35.6% (WWTP-JXZ) of phages in AS systems. This relative broad-spectrum capability allows CRISPR-carrying bacteria to acquire significant phage resistance at a relatively low adaptive cost, which leads to a consistent increase in CRISPR-Cas and spacer density in complex communities. Moreover, CRISPR not only defends against phages but also targets other MGEs, and the number of spacers targeting plasmids is even higher than those targeting phages [64]. Therefore, it is highly likely that the abundance of CRISPR is also influenced by other MGEs. Due to the lack of studies on temporal patterns of spacer density in other environments, the generalizability of this result should be evaluated, especially given that AS is a highly open microbial community, where the enrichment of the adaptive CRISPR-Cas DS might be driven by the frequent invasion of emerging phages and other MGEs introduced with wastewater [65]. Although focus was primarily put on CRISPR-Cas in this study, we recognize that RM also plays a critical role in bacterial defense and gene transfer, particularly in the environments where horizontal gene transfer plays a significant role [66], and the high abundance of RM observed in this study supports its importance.

The observed synchronization between DS and phage dynamics reflects not only to the selection of resistance when phage abundance increases or new phages emerge, but also the reduction in DS abundance and the loss of corresponding CRISPR spacers when phage abundance decreases or phages disappear. This raises a seemingly paradoxical fact: despite the widespread distribution of phages in various environments [1–4], phage defenses do not persistently accumulate within bacterial communities. In fact, both high and low phage-resistant strains of the same species maintained within the community. This contradiction can be reconciled when considering the trade-off between phage resistance and adaptive costs [30]. Studies have shown that stronger phage resistance, whether at the community [67] or individual [68–71] level, often leads to reduced growth rates. Therefore, when phage pressure decreases, communities are quickly selected for low phage resistance but high growth rates. Similarly, this also explains the positively correlation between BOD and DS abundance; when environmental nutrients are more abundant (i.e. higher BOD), interspecies competition among bacteria decreases, leading to the selection of more DSs (higher DS abundance). Furthermore, this selection likely occurs at the strain level. First, consistent with the pan-immunity hypothesis, we observed frequent shifts in strain composition within the relatively stable species-level communities in AS. Second, recent studies have shown that DS genes account for ~90% of the flexible genome in closely related strains of *Vibrio*, *Listeria*, *Salmonella*, and *Clostridium* [72]. In fact, for any species within a complex natural community, maintaining stable colonization requires both defensive capabilities and competitive fitness [73], and even both must be able to adjust at any time to adapt to fluctuations in external phage stress and nutritional restrictions. However, due to the trade-off between phage resistance and fitness cost, a single strain cannot meet such requirements. In this context, increasing strain diversity within a species, with varying levels of phage resistance, is almost the only solution to ensure the stable persistence of the species. Moreover, including our AS data (Supplementary Fig. S11), DSs and MGEs frequently co-occurred in both the isolated bacterial genomes and metagenomic data [74, 75], supporting that there is a high proportion of DS genes in the flexible genome. These genomic observations suggest that interactions between phages and bacteria, mediated through DS, may be one of the primary drivers of strain-level fluctuations in bacterial communities [72]. Therefore, the synchronized variation in DS abundance and phage abundance observed in this study may result from the selective pressure exerted by fluctuating phage abundance on bacterial strains with different levels of phage resistance. From this perspective, it is not surprising that when observing the microbial communities of complex environments at the species level rather than the strain level, the turnover rate of phages is higher than that of bacteria [19, 20].

The pan-immunity model also provides a well explanation for why complex communities exhibit greater phage resistance compared with experimental model communities. The answer may lie in the fact that the synthetic microbial communities used in model experiments typically consist of very few or even single strains per species, which precludes the construction of an effective pan-immune system. Consequently, strain-level community changes are directly reflected by changes at the species level. These results suggest that strain diversity (micro-diversity) within bacterial communities could be a key factor influencing their resistance to phages. Therefore, using phages to manipulate bacterial communities in high-micro-diversity systems (such as AS) may present more challenges compared to low-micro-diversity systems (such as clinical phage therapy [76] and gut phage transplants [46]). In addition, the micro-diversity of synthetic microbiomes can also be artificially increased to enhance the stability and colonization of these communities or microbial agents in actual environments containing phages. However, due to technical limitations, it remains challenging to accurately link phage abundance, host strain abundance, and the number of DSs carried by the host strains within natural communities. In this line, an intriguing next step would be to explore the effect of strain diversity on the stability of bacterial communities against phages in model experiments to further elucidate differences in the conclusions of reductionist and holistic studies.

## Supplementary Material

Supplementary_figures-ISME-20250312_wraf051

Supplementary_tables-ISME-20250312_wraf051

## Data Availability

The metagenomic data were uploaded to the NCBI with an accession number of PRJNA1149857. The public metagenomic data of Shatin WWTP in Hong Kong are available under NCBI accession number PRJNA432264.

## Code availability

Scripts supporting all key analyses of this work are publicly available at https://github.com/Qifeng-Zhang/AS-defense-system.

## References

[1] Dion MB, Oechslin F, Moineau S. Phage diversity, genomics and phylogeny. *Nat Rev Microbiol* 2020;18:125–38. 10.1038/s41579-019-0311-5 32015529

[2] Fan X, Ji M, Mu D et al. Global diversity and biogeography of DNA viral communities in activated sludge systems. *Microbiome* 2023;11:234. 10.1186/s40168-023-01672-1 37865788 PMC10589946

[3] Hoyles L, McCartney AL, Neve H et al. Characterization of virus-like particles associated with the human faecal and caecal microbiota. *Res Microbiol* 2014;165:803–12. 10.1016/j.resmic.2014.10.006 25463385

[4] Gao S-M, Fei H-L, Li Q et al. Eco-evolutionary dynamics of gut phageome in wild gibbons (Hoolock tianxing) with seasonal diet variations. *Nat Commun* 2024;15:1254. 10.1038/s41467-024-45663-8 38341424 PMC10858875

[5] Mushegian AR . Are there 10^31^ virus particles on earth, or more, or fewer? *J Bacteriol* 2020;202:e00052–20. 10.1128/jb.00052-20 PMC7148134 32071093

[6] Wang Y, Ye J, Ju F et al. Successional dynamics and alternative stable states in a saline activated sludge microbial community over 9 years. *Microbiome* 2021;9:199. 10.1186/s40168-021-01151-5 34615557 PMC8495973

[7] Fierer N . Embracing the unknown: disentangling the complexities of the soil microbiome. *Nat Rev Microbiol* 2017;15:579–90. 10.1038/nrmicro.2017.87 28824177

[8] Chevallereau A, Pons BJ, van Houte S et al. Interactions between bacterial and phage communities in natural environments. *Nat Rev Microbiol* 2022;20:49–62. 10.1038/s41579-021-00602-y 34373631

[9] Suttle CA . Marine viruses — major players in the global ecosystem. *Nat Rev Microbiol* 2007;5:801–12. 10.1038/nrmicro1750 17853907

[10] Kiani AK, Anpilogov K, Dautaj A et al. Bacteriophages in food supplements obtained from natural sources. *Acta Biomedica Atenei Parmensis* 2020;91:e2020025. 10.23750/abm.v91i13-S.10834 PMC8023131 33170168

[11] Hsu BB, Gibson TE, Yeliseyev V et al. Dynamic modulation of the gut microbiota and metabolome by bacteriophages in a mouse model. *Cell Host Microbe* 2019;25:803–14.e5. 10.1016/j.chom.2019.05.001 31175044 PMC6579560

[12] Khan Mirzaei M, Deng L. New technologies for developing phage-based tools to manipulate the human microbiome. *Trends Microbiol* 2022;30:131–42. 10.1016/j.tim.2021.04.007 34016512

[13] Alseth EO, Custodio R, Sundius SA et al. The impact of phage and phage resistance on microbial community dynamics. *PLoS Biol* 2024;22:e3002346. 10.1371/journal.pbio.3002346 38648198 PMC11034675

[14] Michael SS, Ian H, Jed AF. Viral effects on bacterial community composition in marine plankton microcosms. *Aquat Microb Ecol* 2004;34:117–27. 10.3354/ame034117

[15] Zhang Z, Zhao H, Mou S et al. Phage infection benefits marine diatom *Phaeodactylum tricornutum* by regulating the associated bacterial community. *Microb Ecol* 2023;86:144–53. 10.1007/s00248-022-02045-1 35622094

[16] Wilde J, Boyes R, Robinson AV et al. Assessing phage-host population dynamics by reintroducing virulent viruses to synthetic microbiomes. *Cell Host Microbe* 2024;32:768–78.e9. 10.1016/j.chom.2024.04.001 38653241

[17] Winter C, Smit A, Herndl GJ et al. Impact of virioplankton on archaeal and bacterial community richness as assessed in seawater batch cultures. *Appl Environ Microbiol* 2004;70:804–13. 10.1128/AEM.70.2.804-813.2004 14766558 PMC348926

[18] Braga LPP, Spor A, Kot W et al. Impact of phages on soil bacterial communities and nitrogen availability under different assembly scenarios. *Microbiome* 2020;8:52. 10.1186/s40168-020-00822-z 32252805 PMC7137350

[19] Needham DM, Chow C-ET, Cram JA et al. Short-term observations of marine bacterial and viral communities: patterns, connections and resilience. *ISME J* 2013;7:1274–85. 10.1038/ismej.2013.19 23446831 PMC3695287

[20] Liu W, Xu C, Li T et al. Temporal dynamics and contribution of phage community to the prevalence of antibiotic resistance genes in a full-scale sludge anaerobic digestion plant. *Environ Sci Technol* 2024;58:6296–304. 10.1021/acs.est.4c00712 38556999

[21] Castledine M, Buckling A. Critically evaluating the relative importance of phage in shaping microbial community composition. *Trends Microbiol* 2024;32:957–69. 10.1016/j.tim.2024.02.014 38604881

[22] Georjon H, Bernheim A. The highly diverse antiphage defence systems of bacteria. *Nat Rev Microbiol* 2023;21:686–700. 10.1038/s41579-023-00934-x 37460672

[23] Doron S, Melamed S, Ofir G et al. Systematic discovery of antiphage defense systems in the microbial pangenome. *Science* 2018;359:eaar4120. 10.1126/science.aar4120 29371424 PMC6387622

[24] Tesson F, Herve A, Mordret E et al. Systematic and quantitative view of the antiviral arsenal of prokaryotes. *Nat Commun* 2022;13:2561. 10.1038/s41467-022-30269-9 35538097 PMC9090908

[25] Beavogui A, Lacroix A, Wiart N et al. The defensome of complex bacterial communities. *Nat Commun* 2024;15:2146. 10.1038/s41467-024-46489-0 38459056 PMC10924106

[26] Huang D, Yuan MM, Chen J et al. The association of prokaryotic antiviral systems and symbiotic phage communities in drinking water microbiomes. *ISME Commun* 2023;3:46. 10.1038/s43705-023-00249-1 37142716 PMC10160068

[27] Somerville V, Schowing T, Chabas H et al. Extensive diversity and rapid turnover of phage defense repertoires in cheese-associated bacterial communities. *Microbiome* 2022;10:137. 10.1186/s40168-022-01328-6 36028909 PMC9419375

[28] Hochhauser D, Millman A, Sorek R. The defense island repertoire of the *Escherichia coli* pan-genome. *PLoS Genet* 2023;19:e1010694. 10.1371/journal.pgen.1010694 37023146 PMC10121019

[29] Costa AR, van den Berg DF, Esser JQ et al. Accumulation of defense systems in phage-resistant strains of *Pseudomonas aeruginosa*. *Sci Adv* 2024;10:eadj0341. 10.1126/sciadv.adj0341 38394193 PMC10889362

[30] Bernheim A, Sorek R. The pan-immune system of bacteria: antiviral defence as a community resource. *Nat Rev Microbiol* 2020;18:113–9. 10.1038/s41579-019-0278-2 31695182

[31] Kershaw GB . The activated sludge process. *Nature* 1928;121:165–6. 10.1038/121165a0

[32] Daims H, Taylor MW, Wagner M. Wastewater treatment: a model system for microbial ecology. *Trends Biotechnol* 2006;24:483–9. 10.1016/j.tibtech.2006.09.002 16971007

[33] Zhang J, Tang A, Jin T et al. A panoramic view of the virosphere in three wastewater treatment plants by integrating viral-like particle-concentrated and traditional non-concentrated metagenomic approaches. *iMeta* 2024;3:e188. 10.1002/imt2.188 38898980 PMC11183165

[34] Yin X, Deng Y, Ma L et al. Exploration of the antibiotic resistome in a wastewater treatment plant by a nine-year longitudinal metagenomic study. *Environ Int* 2019;133:105270. 10.1016/j.envint.2019.105270 31683155

[35] Bolger AM, Lohse M, Usadel B. Trimmomatic: a flexible trimmer for Illumina sequence data. *Bioinformatics* 2014;30:2114–20. 10.1093/bioinformatics/btu170 24695404 PMC4103590

[36] Li D, Liu C-M, Luo R et al. MEGAHIT: an ultra-fast single-node solution for large and complex metagenomics assembly via succinct de Bruijn graph. *Bioinformatics* 2015;31:1674–6. 10.1093/bioinformatics/btv033 25609793

[37] Parks DH, Imelfort M, Skennerton CT et al. CheckM: assessing the quality of microbial genomes recovered from isolates, single cells, and metagenomes. *Genome Res* 2015;25:1043–55. 10.1101/gr.186072.114 25977477 PMC4484387

[38] Li H . Aligning sequence reads, clone sequences and assembly contigs with BWA-MEM. arXiv 2013; arXiv:1303.3997. 10.48550/arXiv.1303.3997

[39] Hyatt D, Chen G-L, LoCascio PF et al. Prodigal: prokaryotic gene recognition and translation initiation site identification. *BMC Bioinf* 2010;11:119. 10.1186/1471-2105-11-119 PMC2848648 20211023

[40] Uritskiy GV, DiRuggiero J, Taylor J. MetaWRAP—a flexible pipeline for genome-resolved metagenomic data analysis. *Microbiome* 2018;6:158. 10.1186/s40168-018-0541-1 30219103 PMC6138922

[41] Chaumeil P-A, Mussig AJ, Hugenholtz P et al. GTDB-Tk: a toolkit to classify genomes with the genome taxonomy database. *Bioinformatics* 2019;36:1925–7. 10.1093/bioinformatics/btz848 31730192 PMC7703759

[42] Jain C, Rodriguez-R LM, Phillippy AM et al. High throughput ANI analysis of 90K prokaryotic genomes reveals clear species boundaries. *Nat Commun* 2018;9:5114. 10.1038/s41467-018-07641-9 30504855 PMC6269478

[43] Asnicar F, Thomas AM, Beghini F et al. Precise phylogenetic analysis of microbial isolates and genomes from metagenomes using PhyloPhlAn 3.0. *Nat Commun* 2020;11:2500. 10.1038/s41467-020-16366-7 32427907 PMC7237447

[44] Wood DE, Lu J, Langmead B. Improved metagenomic analysis with kraken 2. *Genome Biol* 2019;20:257. 10.1186/s13059-019-1891-0 31779668 PMC6883579

[45] Lu J, Breitwieser FP, Thielen P et al. Bracken: estimating species abundance in metagenomics data. *PeerJ Comput Sci* 2017;3:e104. 10.7717/peerj-cs.104 PMC12016282 40271438

[46] Truong DT, Tett A, Pasolli E et al. Microbial strain-level population structure and genetic diversity from metagenomes. *Genome Res* 2017;27:626–38. 10.1101/gr.216242.116 28167665 PMC5378180

[47] Guo J, Bolduc B, Zayed AA et al. VirSorter2: a multi-classifier, expert-guided approach to detect diverse DNA and RNA viruses. *Microbiome* 2021;9:37. 10.1186/s40168-020-00990-y 33522966 PMC7852108

[48] Kieft K, Zhou Z, Anantharaman K. VIBRANT: automated recovery, annotation and curation of microbial viruses, and evaluation of viral community function from genomic sequences. *Microbiome* 2020;8:90. 10.1186/s40168-020-00867-0 32522236 PMC7288430

[49] Nayfach S, Camargo AP, Schulz F et al. CheckV assesses the quality and completeness of metagenome-assembled viral genomes. *Nat Biotechnol* 2021;39:578–85. 10.1038/s41587-020-00774-7 33349699 PMC8116208

[50] Fu L, Niu B, Zhu Z et al. CD-HIT: accelerated for clustering the next-generation sequencing data. *Bioinformatics* 2012;28:3150–2. 10.1093/bioinformatics/bts565 23060610 PMC3516142

[51] Roux S, Adriaenssens EM, Dutilh BE et al. Minimum information about an uncultivated virus genome (MIUViG). *Nat Biotechnol* 2019;37:29–37. 10.1038/nbt.4306 30556814 PMC6871006

[52] Beghini F, McIver LJ, Blanco-Míguez A et al. Integrating taxonomic, functional, and strain-level profiling of diverse microbial communities with bioBakery 3. *eLife* 2021;10:e65088. 10.7554/eLife.65088 33944776 PMC8096432

[53] Consortium TU . UniProt: a worldwide hub of protein knowledge. *Nucleic Acids Res* 2018;47:D506–15. 10.1093/nar/gky1049 PMC6323992 30395287

[54] Caspi R, Billington R, Fulcher CA et al. The MetaCyc database of metabolic pathways and enzymes. *Nucleic Acids Res* 2017;46:D633–9. 10.1093/nar/gkx935 PMC5753197 29059334

[55] Skennerton CT, Imelfort M, Tyson GW. Crass: identification and reconstruction of CRISPR from unassembled metagenomic data. *Nucleic Acids Res* 2013;41:e105–5. 10.1093/nar/gkt183 23511966 PMC3664793

[56] McGinnis S, Madden TL. BLAST: at the core of a powerful and diverse set of sequence analysis tools. *Nucleic Acids Res* 2004;32:W20–5. 10.1093/nar/gkh435 15215342 PMC441573

[57] Brown CL, Mullet J, Hindi F et al. mobileOG-db: a manually curated database of protein families mediating the life cycle of bacterial mobile genetic elements. *Appl Environ Microbiol* 2022;88:e00991–22. 10.1128/aem.00991-22 36036594 PMC9499024

[58] The State Environmental Protection Administration . Water and Wastewater Monitoring and Analysis Methods. Beijing: China Environmental Science Press, 2002.

[59] Wang Y, Jiang X, Liu L et al. High-resolution temporal and spatial patterns of virome in wastewater treatment systems. *Environ Sci Technol* 2018;52:10337–46. 10.1021/acs.est.8b03446 30148618

[60] Hernandez CA, Koskella B. Phage resistance evolution in vitro is not reflective of in vivo outcome in a plant-bacteria-phage system. *Evolution* 2019;73:2461–75. 10.1111/evo.13833 31433508

[61] Blazanin M, Turner PE. Community context matters for bacteria-phage ecology and evolution. *ISME J* 2021;15:3119–28. 10.1038/s41396-021-01012-x 34127803 PMC8528888

[62] Hille F, Charpentier E. CRISPR-Cas: biology, mechanisms and relevance. *Philos Trans Roy Soc B Biol Sci* 2016;371:20150496. 10.1098/rstb.2015.0496 PMC5052741 27672148

[63] Stoltzfus MJ, Workman RE, Keith NC et al. A dynamic subpopulation of CRISPR–Cas overexpressers allows *streptococcus pyogenes* to rapidly respond to phage. *Nat Microbiol* 2024;9:2410–21. 10.1038/s41564-024-01748-0 38997519 PMC11983678

[64] Martínez Arbas S, Narayanasamy S, Herold M et al. Roles of bacteriophages, plasmids and CRISPR immunity in microbial community dynamics revealed using time-series integrated meta-omics. *Nat Microbiol* 2021;6:123–35. 10.1038/s41564-020-00794-8 33139880 PMC7752763

[65] Wu Q, Liu W-T. Determination of virus abundance, diversity and distribution in a municipal wastewater treatment plant. *Water Res* 2009;43:1101–9. 10.1016/j.watres.2008.11.039 19095276

[66] Shaw LP, Rocha EPC, MacLean RC. Restriction-modification systems have shaped the evolution and distribution of plasmids across bacteria. *Nucleic Acids Res* 2023;51:6806–18. 10.1093/nar/gkad452 37254807 PMC10359461

[67] Yang JW, Chang F-H, Yeh Y-C et al. Trade-offs between competitive ability and resistance to top-down control in marine microbes. *mSystems* 2023;8:e0101722. 10.1128/msystems.01017-22 36916988 PMC10134844

[68] Liu Z-L, Hu E-Z, Niu D-K et al. Investigating the relationship between CRISPR-Cas content and growth rate in bacteria. *Microbiol Spectrum* 2023;11:e03409–22. 10.1128/spectrum.03409-22 PMC10269591 37022199

[69] Kolan D, Cattan-Tsaushu E, Enav H et al. Tradeoffs between phage resistance and nitrogen fixation drive the evolution of genes essential for cyanobacterial heterocyst functionality. *ISME J* 2024;18:1–16. 10.1093/ismejo/wrad008 PMC10811720 38365231

[70] Meaden S, Paszkiewicz K, Koskella B. The cost of phage resistance in a plant pathogenic bacterium is context-dependent. *Evolution* 2015;69:1321–8. 10.1111/evo.12652 25809535 PMC4979666

[71] Markwitz P, Lood C, Olszak T et al. Genome-driven elucidation of phage-host interplay and impact of phage resistance evolution on bacterial fitness. *ISME J* 2021;16:533–42. 10.1038/s41396-021-01096-5 34465897 PMC8776877

[72] Hussain FA, Dubert J, Elsherbini J et al. Rapid evolutionary turnover of mobile genetic elements drives bacterial resistance to phages. *Science* 2021;374:488–92. 10.1126/science.abb1083 34672730

[73] Våge S, Bratbak G, Egge J et al. Simple models combining competition, defence and resource availability have broad implications in pelagic microbial food webs. *Ecol Lett* 2018;21:1440–52. 10.1111/ele.13122 30014593

[74] Botelho J . Defense systems are pervasive across chromosomally integrated mobile genetic elements and are inversely correlated to virulence and antimicrobial resistance. *Nucleic Acids Res* 2023;51:4385–97. 10.1093/nar/gkad282 37078595 PMC10201445

[75] Rocha EPC, Bikard D. Microbial defenses against mobile genetic elements and viruses: who defends whom from what? *PLoS Biol* 2022;20:e3001514. 10.1371/journal.pbio.3001514 35025885 PMC8791490

[76] Pirnay J-P, Djebara S, Steurs G et al. Personalized bacteriophage therapy outcomes for 100 consecutive cases: a multicentre, multinational, retrospective observational study. *Nat Microbiol* 2024;9:1434–53. 10.1038/s41564-024-01705-x 38834776 PMC11153159

