# Supplementary material for: Long-term metagenomic insights into the roles of antiviral defense systems in stabilizing activated sludge bacterial communities
Source: ISME J. 2025 Mar 17;19(1):wraf051. doi: 10.1093/ismejo/wraf051 (PMC11980602; doi:10.1093/ismejo/wraf051)
Supplement: Supplementary_figures-ISME-20250312_wraf051 [file supplementary_figures-isme-20250312_wraf051.pdf]

Title:

Long-term metagenomic insights into the roles of antiviral defense systems in stabilizing activated sludge bacterial communities

Running Title:

Defense systems in bacterial communities

Authors:

Qifeng Zhang, Jie Li, Jinhua Tuo, Shengnan Liu, Yang Liu, Peng Liu*, Lin Ye, Xu-Xiang Zhang*

Affiliations:

State Key Laboratory of Pollution Control and Resource Reuse, School of Environment, Nanjing University, Nanjing 210023, China

***Corresponding author:**

Peng Liu: State Key Laboratory of Pollution Control and Resource Reuse, School of Environment, Nanjing University, Nanjing 210023, China

Email address: lp085853@163.com

Xu-Xiang Zhang: State Key Laboratory of Pollution Control and Resource Reuse, School of Environment, Nanjing University, Nanjing 210023, China

Email address: zhangxx@nju.edu.cn

Supplementary Information

13 Pages including cover page

11 Figures

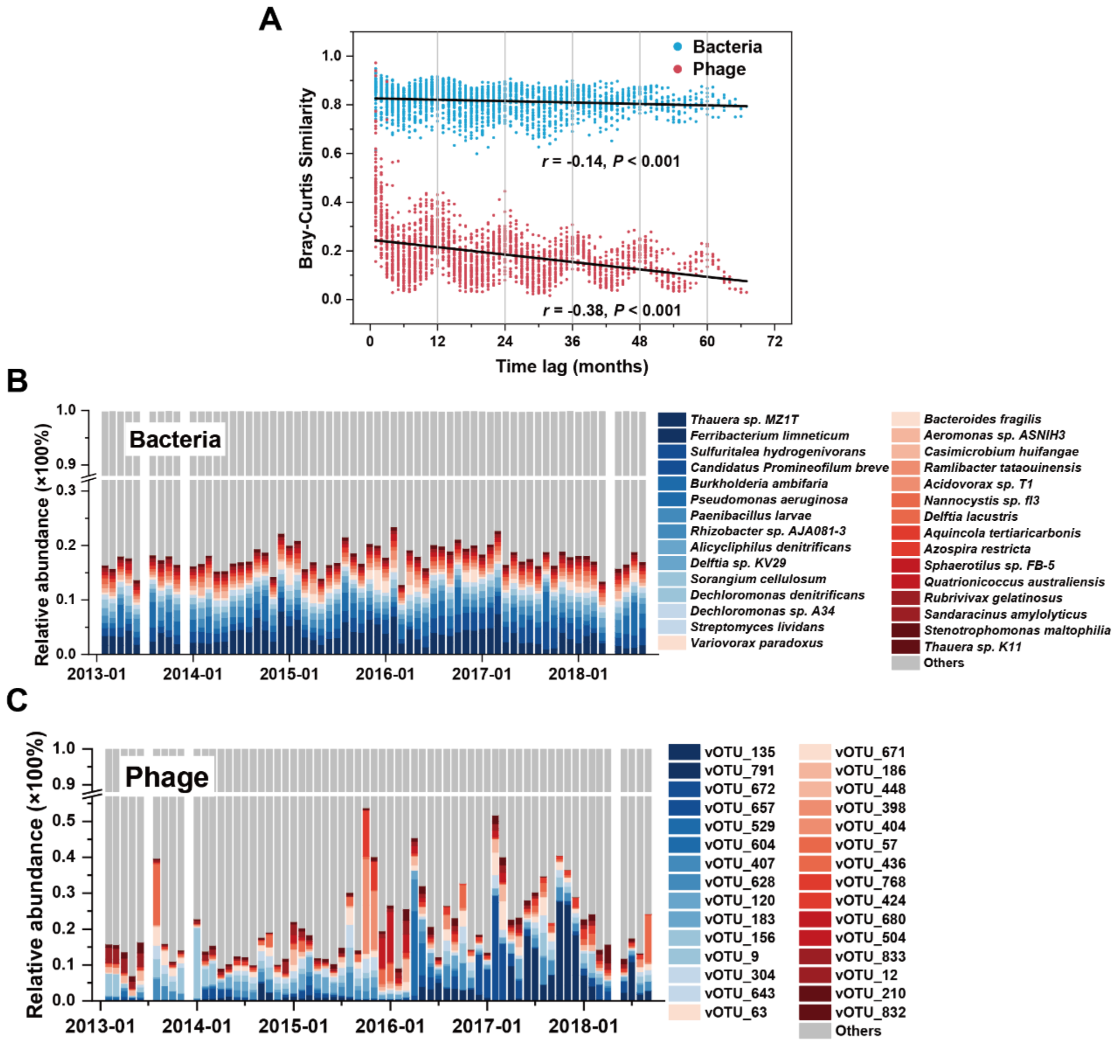

Fig. S1: Temporal dynamics of bacterial and phage community in WWTP-JXZ.

A Bray-Curtis similarity of bacterial and phage communities over different time lags, with each point representing a sample pair, encompassing all possible sample pairs (n = 2346). **B** Relative abundance changes of bacterial species, showing the top 30 most abundant species. **C** Relative abundance changes of phage species, showing the top 30 most abundant species.

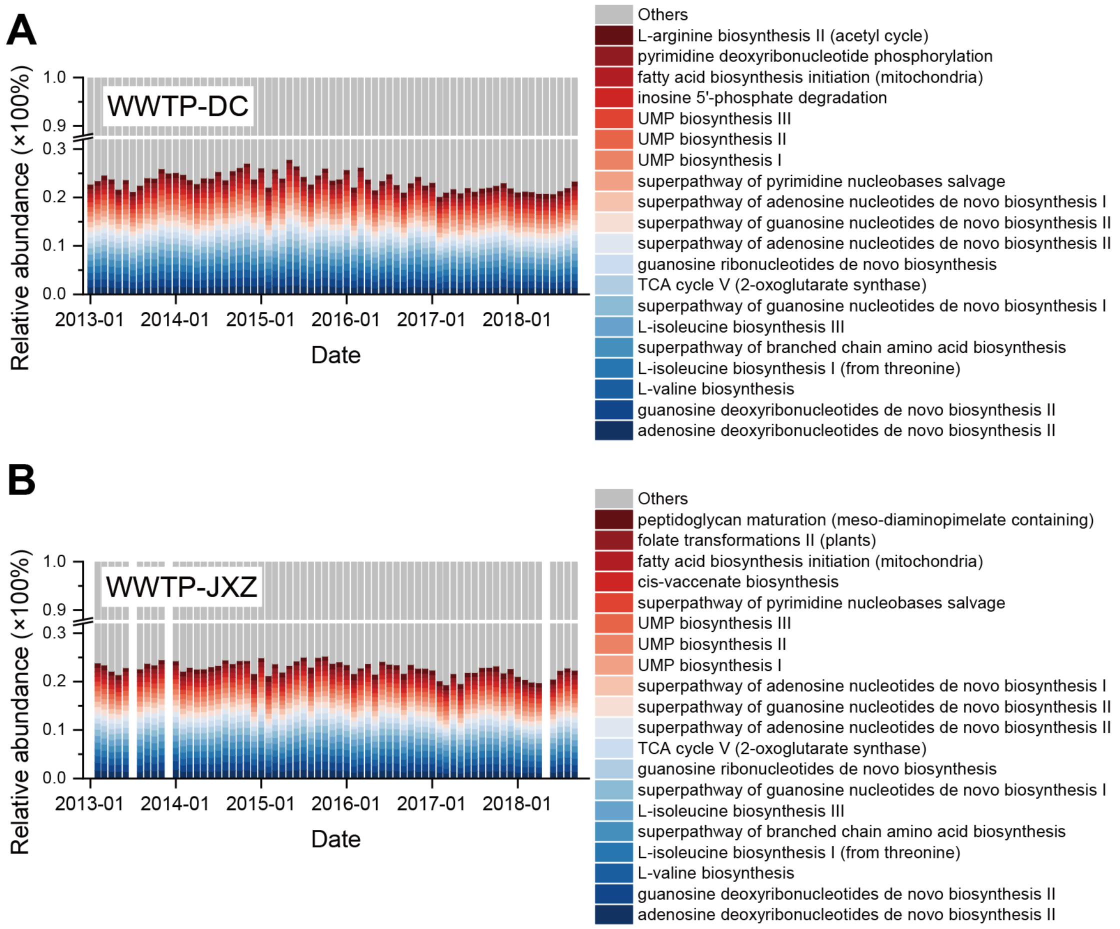

Fig. S2: Temporal dynamics of bacterial community function. A WWTP-DC. **B** WWTP-JXZ. Only the top 20 most abundant MetaCyc pathways are displayed.

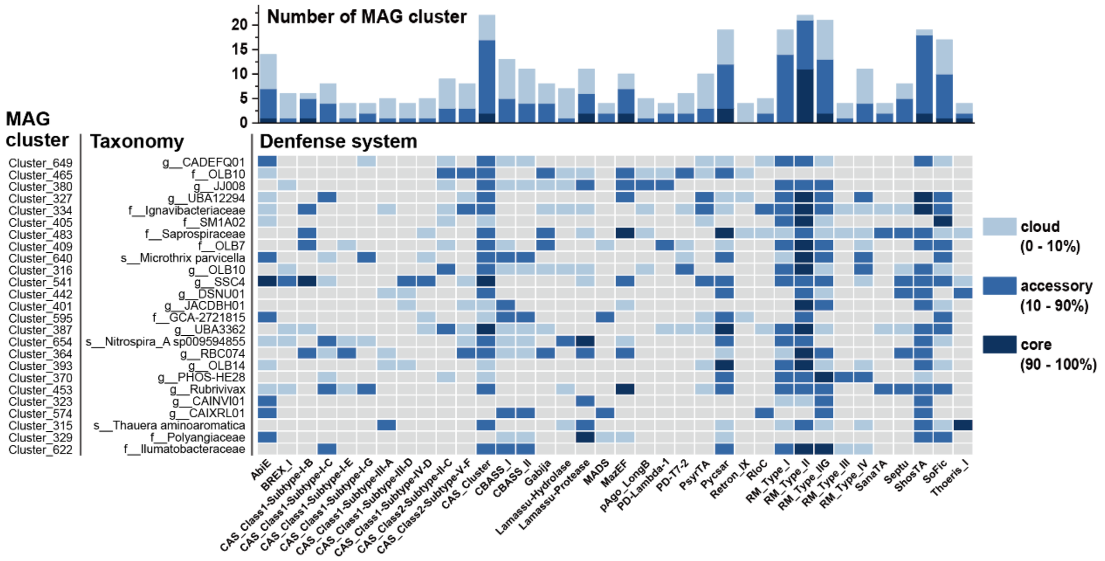

Fig. S3: Taxon-specific DS repertoires. Frequency of DSs in MAG clusters (n = 25) with more than 20 members (ANI ≥ 95%). DSs are classified based on frequency as core (> 90% of MAGs), accessory (90% < of MAGs > 10%), and cloud (< 10% of MAGs). The left side shows the GTDB species annotations of MAGs, and the top side shows the number of MAG clusters for each DS.

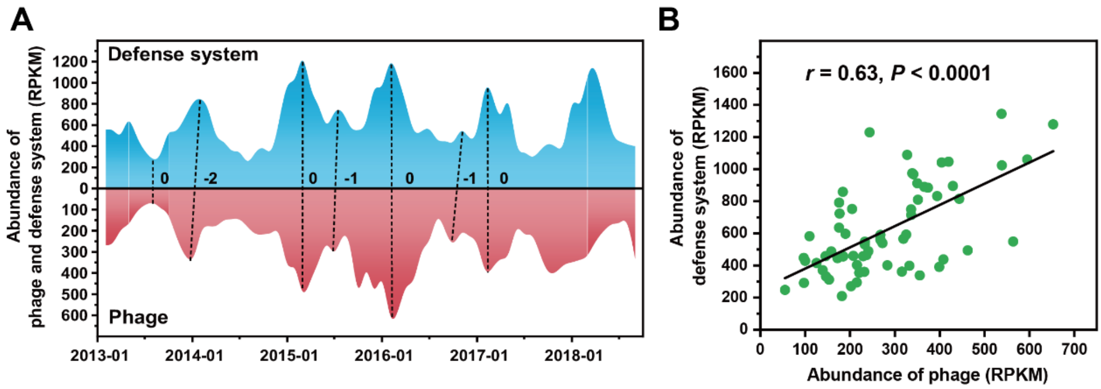

Fig. S4: Temporal dynamics of DSs in AS of WWTP-JXZ. A Temporal changes in DS and phage abundances. Considering that a single defense gene may not be effective, we defined the abundance of DSs as the abundance of contigs containing complete DSs. **B** Linear regression between DS abundance and phage abundance.

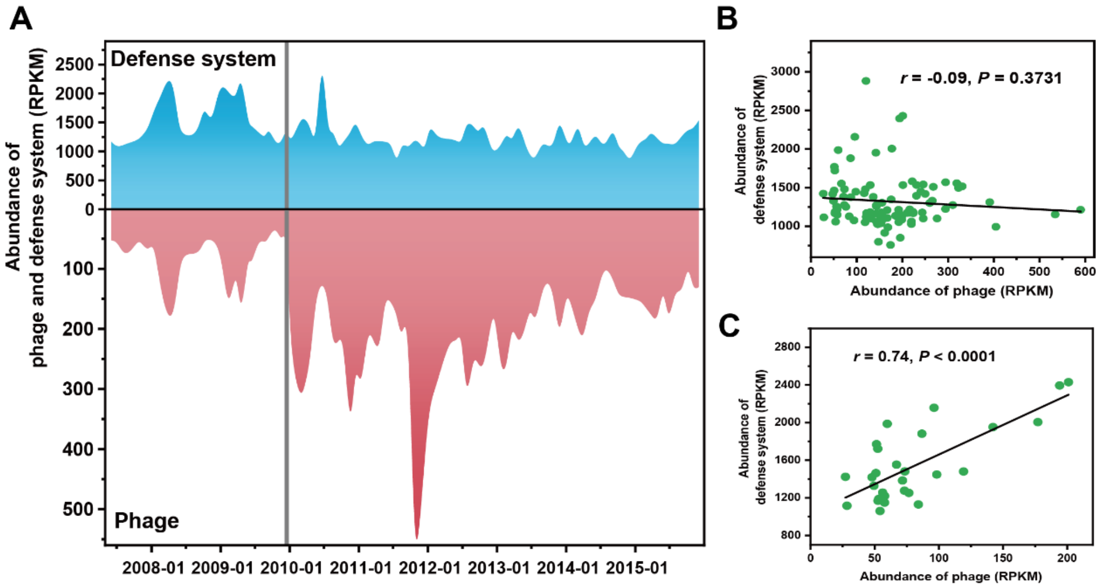

Fig. S5: Temporal dynamics of DS in AS of WWTP-HK. A Temporal changes in DS and phage abundances. Considering that a single defense gene may not be effective, we defined the abundance of DSs as the abundance of contigs containing complete DSs. **B** Linear regression between DS abundance and phage abundance (2007-2016). **C** Linear regression between DS abundance and phage abundance during the first three stable years of operation (2007-2010).

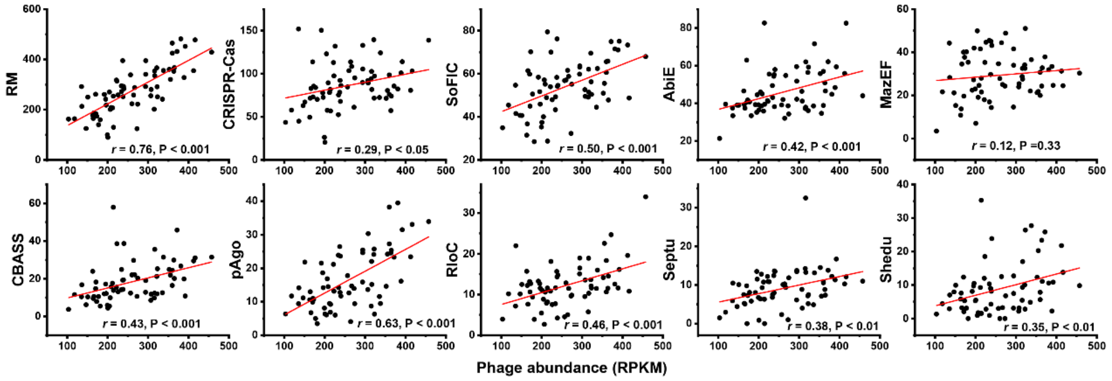

Fig. S6: Correlation between the abundances of TOP 10 DSs and total phage abundance. The x-axis is the total abundance of phages, and the y-axis is the abundance of DS.

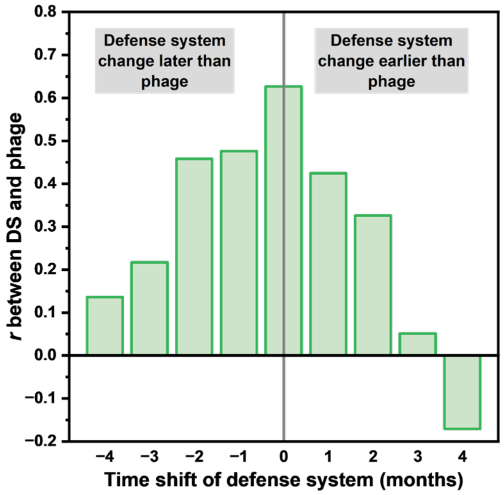

Fig. S7: Time shift correlation between DS abundance and phage abundance in WWTP-JXZ. The x-axis values indicate the time shift of the DS, e.g., -1 represents the correlation between the phage abundance of each month and the DS abundance of the following month.

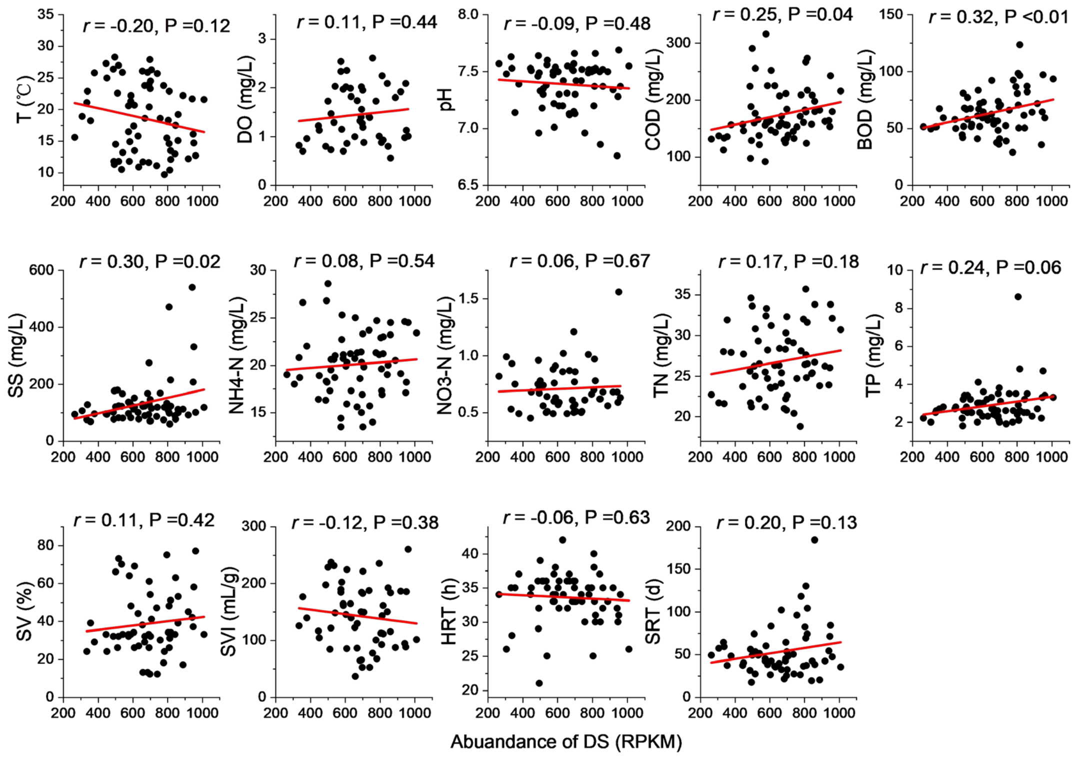

Fig. S8: Correlation between the abundances of DS and water physicochemical properties and process parameters. The x-axis represents the abundance of DS, and the y-axis represents water physicochemical properties and process parameters.

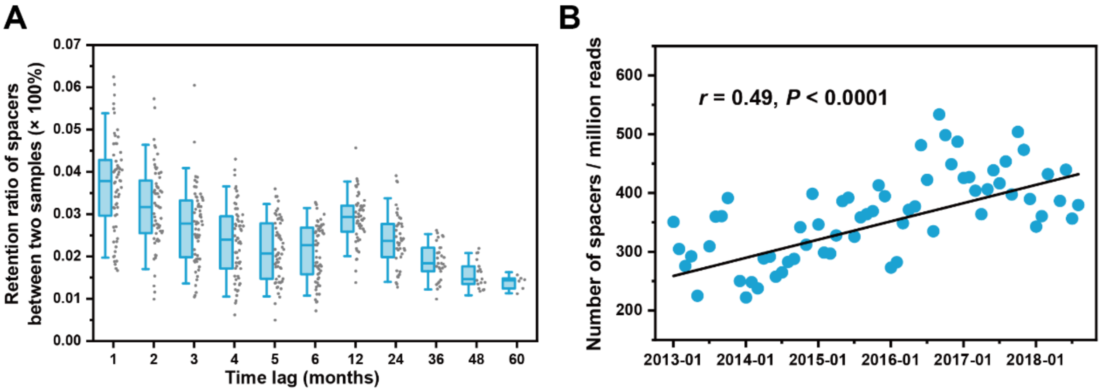

Fig. S9: Temporal dynamics of CRISPR spacer in AS of WWTP-JXZ. A Retention ratio of CRISPR spacers at different time lags, with each point representing a sample pair. **B** Linear regression between CRISPR spacer density and time.

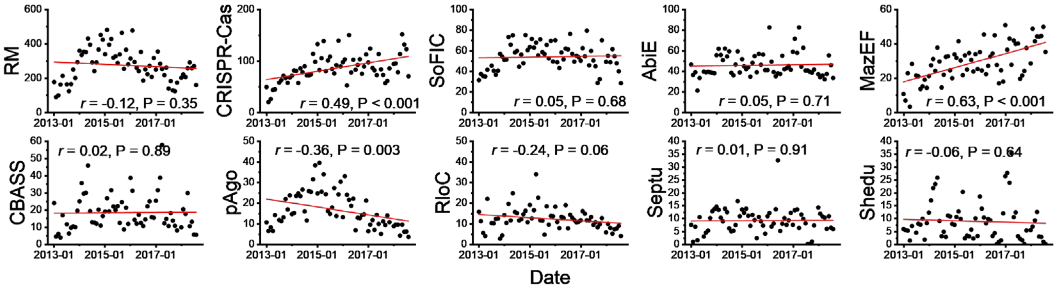

Fig. S10: Correlation between the abundances of TOP 10 DSs and time. The x-axis is date, and the y-axis is the abundance of DS.

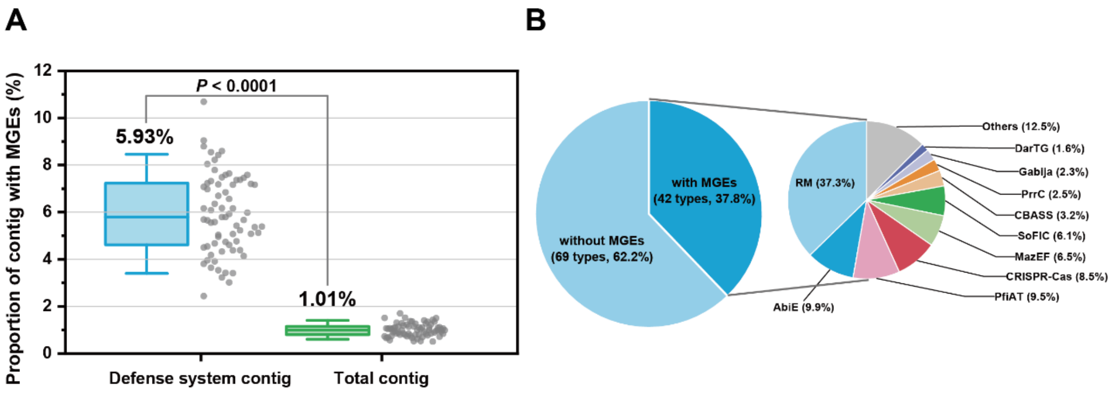

Fig. S11: Co-occurrence of DSs and MGEs. A Proportion of DS contigs and total contigs that contain MGEs. **B** Types of DS co-located with MGEs.
